# Variations of circulating endothelial progenitor cells and transforming growth factor-beta-1 (TGF-β1) during thoracic radiotherapy are predictive for radiation pneumonitis

**DOI:** 10.1186/1748-717X-8-189

**Published:** 2013-07-25

**Authors:** Yunfang Liu, Tingyi Xia, Wenjun Zhang, Yongjie Zhong, Luhua Zhang, Xuan Wang, Huiming Yu

**Affiliations:** 1 Department of Diagnosis, Shandong University Medical School, Jinan, Shandong Province 250012, China; 2 Cancer Center, Air Force General Hospital, PLA, Beijing 100012, China; 3 Department of Radiotherapy, Capital Medical University Affiliated Beijing Chao- yang Hospital, Beijing 100020, China; 4 Present address: Huiming Yu, Department of Radiotherapy, Capital Medical University Affiliated Beijing Chao-yang Hospital, No. 8, Gongti South Road, Beijing 100020, P.R. China

**Keywords:** Non-small cell lung cancer, Radiation pneumonitis, Transforming growth factor-beta-1, Endothelial progenitor cells

## Abstract

**Background:**

The vascular endothelial cells are important targets of radiotherapy, which may be involved in the pathogenesis of radiation pneumonitis (RP). This study investigated the variations of circulating endothelial progenitor cells (EPCs) and transforming growth factor-beta-1 (TGF-β1) during three-dimensional conformal radiation therapy (3D-CRT) in patients with non–small-cell lung cancer (NSCLC) and analyzed the correlation between these variations with the occurrence of RP.

**Patients and methods:**

From November 2008 to November 2009, eighty-four consecutive patients receiving 3D-CRT for stage III disease were evaluated prospectively. Circulating EPCs and TGF-β1 levels were measured at baseline, every 2 weeks during, and at the end of treatment. RP was evaluated prospectively at 6 weeks after 3D-CRT.

**Results:**

Thirty-eight patients (47.5%) experienced score 1 or more of RP. The baseline levels of EPCs and TGF-β1 were analyzed, no difference was found between patients with and without RP during and after 3D-CRT. By serial measurement of TGF-β1 and EPCs levels, we found that the mean levels of EPCs in the whole population remained stable during radiotherapy, but the mean levels of TGF-β1 increased slowly during radiotherapy. TGF-β1 and EPCs levels were all significantly higher at week 2, week 4 and week 6 in patients with RP than that in patients without RP, respectively. During the period of radiation treatment, TGF-β1 levels began to increase in the first 2 weeks and became significantly higher at week 6 (*P* < 0.01). EPCs levels also began to increase in the first 2 weeks and reached a peak at week 4. Using an ANOVA model for repeated-measures, we found significant associations between the levels of TGF-β1 and EPCs during the course of 3D-CRT and the risk of developing RP (*P* < 0.01). Most of the dosimetric factors showed a significant association with RP.

**Conclusion:**

Early variations of TGF-β1 and EPCs levels during 3D-CRT are significantly associated with the risk of RP. Variations of circulating TGF-β1 and EPCs levels during 3D-CRT may serve as independent predictive factors for RP.

**Trial registration:**

Trials registration number: 20070618

## Background

Non–small-cell lung cancer (NSCLC) is the most common cancer in developed countries. For patients with locally advanced or medically inoperable disease, radiotherapy has been an important intervention method. Radiation pneumonitis (RP) is the acute expression of radio-induced ung damage. Among the patients received thoracic radiotherapy, 8% to 13% developed severe toxicity, and approximately 1.6% died from RP [1]. However, the underlying molecular and cellular mechanisms of RP are very complex. Several biological factors need to be considered for understanding the molecular events in developing radiation-induced complications in normal tissues [2-5].

Radiotherapy is an important nonsurgical treatment for cancer. Recent studies have shown that tumor vasculature and, in particular, the vascular endothelial cells are important targets of radiotherapy [6,7], which may be involved in the pathogenesis of RP [8]. The damage of vascular endothelial cells may increase the infiltration of inflammatory cells into the pulmonary interstitium and alveolar. Some believe that damage to vascular endothelial cells plays a critical role in increasing the levels of endothelial progenitor cells (EPCs) [9]. EPCs as well as mature endothelial cells are detectable in the peripheral circulation [10]. EPCs may appear in the circulation by detaching from activated or damaged vessels. An increase of circulating EPCs was described in several pathologic conditions that involve vascular injury or instability as myocardial infarction and cancer [11,12]. Emerging evidence suggests that circulating EPCs may provide an endogenous repair mechanism to counteract ongoing risk factor-induced endothelial injury and therefore protect against the development of RP [13]. Therefore, we postulated that the changes in EPCs levels may predict the incidence of RP.

Transforming growth factor-beta-1 (TGF-β1) is a pleiotropic cytokine that has been found to be highly associated with the damage of lung architecture. TGF-β1 can regulate tissue morphogenesis and inhibit the proliferation of many cell types [14], which may play an important role in the pathogenesis of RP [15]. Several recent studies have proved that radiation induces the expression of TGF-β1 on vascular endothelial cells [16]. TGF-β1 has multiple effects on vascular endothelial cells. In vivo, TGF-β1 is one of the key stimuli of endothelial proliferation and migration and thus plays an essential role in physiological and pathological angiogenesis [17,18]. Notably, TGF-β1 stimulates directly the proliferation of fibroblasts and endothelial cell [19]. Previous study proved that TGF-β1 induces angiogenesis in vivo through an indirect mechanism, by up-regulating the expression of VEGF in epithelial or other cell types [20]. Thus, TGF-β1 activity appears to be necessary to the loss of lung architecture.

We hypothesized that radiotherapy might increase the mobilization of EPCs by congregating on the damaged vessels. This study was therefore designed to determine the changes in circulating EPCs and TGF-β1 during three-dimensional conformal radiation therapy (3D-CRT) in patients with NSCLC and to analyze the correlation between these variations with the occurrence of RP.

## Methods

### Patient eligibility

This study was approved by the local ethics committee, and informed consent was obtained from all study participants. From November 2008 to November 2009, eighty-four consecutive patients were enrolled in this prospective study. Criteria for enrollment include: (1) ages > 18 years; (2) locally unresectable stage III NSCLC proven either by biopsy or cytology; (3) life expectancy ≥ 6 months; (4) Karnofsky performance status (KPS) ≥ 80; (5) good pulmonary function tests (ratio of forced expiratory volume at 1 second on vital capacity ≥ 50%, ratio of diffusion capacity for carbon monoxide on alveolar volume ≥50%). At the time of study entry the patients had to be free of additional malignant, inflammatory or ischemic diseases, wounds or ulcers, pulmonary fibrosis that may influence the results of this study.

### Treatment description

As previously reported [21], patients received conventional fractionated RT (2 Gy per fraction, 5 days per week). The total irradiation dose ranged from 60 to 72 Gy, with a median of 66 Gy. Target volumes were defined according to the International Commission on Radiation Units and Measurements (ICRU) -50 report [22]. The structures of interest, such as GTV, clinical target volumes, and normal structures were contoured on multiple computed tomography pictures. Doses were calculated taking into account the tissue density heterogeneity, and dose volume histograms (DVHs) of the lungs were calculated based on computed tomography–defined lung volumes. Total mean lung dose (MLD), the percentage of irradiated lung volume exceeding 20 Gy (V20) and 30 Gy (V30) were calculated from lung DVHs. The total MLD was calculated as follows: MLD = [(right lung volume × mean lung dose to right lung) + (left lung volume × mean lung dose to left lung)]/ (left lung volume + right lung volume). The lung dosimetric factors were calculated with subtraction of the GTV. Both lungs were considered either as a single paired organ or as two separate organs. 3D-CRT treatment planning was performed using the beam’s-eye-view technique. Megavoltage equipment was used with photon energies of 6 or 15 MV using a multileaf collimator to shape the irradiation portals according to the target volume. The first part of radiation used six portal entrances (anterior/posterior, posterior/anterior and 4 oblique beam) for a total prescribed dose of 50 Gy. An additional dose of 10–20 Gy was prescribed using six portal entrances (0°, 51°, 102°, 145°, 215°, and 306°). Six portals were treated each day. All patients were treated with similar medications during the observation period.

### Evaluation of RP

At 6 to 8 weeks after the end of 3D-CRT, the severity of RP was determined using the Lent-Soma scale defined by the Radiation Therapy Oncology Group (RTOG) and the European Organization for the Research and Treatment of Cancer (EORTC) [23]. Thus, RP was scored here from clinical symptoms, radiological abnormalities, and loss of pulmonary function, which include three subjective scales and two objective scales. Subjective scales: cough; dyspnea; thoracic pain. Objective scales: chest x-ray and thoracic CT read by an independent committee of experts (pneumologists, radiologists and radiation oncologists); PF tests (reduction of vital capacity and/or diffusion capacity for carbon monoxide on alveolar volume). All single-scale measures ranged from 0 to 4 in score. The final scoring was equal to the average of the five scores. RP was defined as the grade ≥1.

### Measurement of circulating TGF-β1

Blood samples were collected from patients prior to treatment (baseline), then every 2 weeks during, and at 4 weeks after the end of treatment (i.e., 5 blood samples). Blood was collected in tubes containing 7.5% K3 EDTA and immediately placed on ice. The samples were centrifuged at 4°C for 30 min at 1000 g within 1 h upon collection, and then frozen at −80°C for further analysis. The plasma for TGF-β1 determination was withdrawn from the middle of the plasma column trying to avoid the platelet interface. TGF-β1 concentration in plasma was measured using a specific enzyme immunoassay kit from immunotech (Biosource, USA).

### Isolation and quantification of EPCs by flow cytometry

Blood samples were collected from patients prior to treatment (baseline), then every 2 weeks during, and at 4 weeks after the end of treatment. Monocytes were isolated and purified from 20 ml of peripheral blood. In brief, 20 ml blood diluted 1:1 in phosphate-buffered saline (PBS) was layered on top of lymphoprep density gradient media at 1.077 plus or minus 0.001 g/ml (Pharmacia Biotech, Uppsala, Sweden) to separate peripheral blood mononuclear cells (PBMCs). Tubes were centrifuged for 30 minutes at 400 g. PBMCs on top of the separation media were carefully collected and then washed 3 times with PBS.

CD34+ monocytes were isolated from PBMCs using immunomagnetic CD34 microbeads (Miltenyi direct CD34 progenitor cell isolation kit, Miltenyi Biotech, Bergisch-Gladbach, Germany) as reported previously [24]. After the magnetic labeling of the cells, the suspension was loaded onto a MACS Column (MS Columns, Miltenyi Biotec) which was placed in the magnetic field of a MACS Separator (Octo MACS Cell Separator, Miltenyi Biotec). The magnetically labeled CD34+ cells were retained within the column. After removing the column from the magnetic field, the magnetically- retained CD34+ cells were eluted as a positively-selected cell fraction. The CD34+ cells were counted with the Neubauer counting chamber. After the positive selection, CD34+ MNCs were counted by FACS (fluorecenceactivated cell sorting) analysis. The number of CD34+ cells were quantified and expressed as number of cells per milliliter of blood. All samples were measured in duplicate and the values averaged. For all assays the intra-observer and inter-observer variation coefficient was less than 5%, respectively.

### Statistical analysis

Continuous data were expressed as mean ± SD, and discrete data were given as counts and percentages. A student’s t-test was used to evaluate the relationship between the clinical, functional, and dosimetric factors on RP. Pearson Chi-Square test was used to compare categorical variables, and independent-samples T test or one-way ANOVA were used for quantitative variables, as appropriate. Linear regression analysis was employed to determine the correlation between the changes of EPCs and TGF-β1 in the same patient. In the univariate analysis, the occurrence of RP and potential prognostic features were analyzed with the standard statistical analysis methods. An SPSS software package (version 13.0; SPSS Inc) was adopted for analysis and a value of P < 0.05 was regarded as statistically significant.

## Results

### Patient characteristics

Of the 84 patients enrolled, four patients were excluded from analysis due to incomplete treatment (1 patient), unsatisfactory blood sampling (1 patient) and cancer progression (2 patients). Finally, 80 patients were enrolled in this study including 48 males and 32 females. Their ages ranged from 38 to 70 years, with a mean age of 58.5 years. There were 43 squamous cell carcinomas, 31 adenocarcinomas, 3 large cell carcinomas and 3 others. Seven patients had chronic obstructive pulmonary disease, 3 with cardiovascular disease, and 3 with diabetes. No patients had any evidence of interstitial pulmonary fibrosis. Chemotherapy was done before radiation in 21 patients (26.3%), and concurrent and post chemotherapy was done in 35 patients (43.8%). The used regimens for concurrent chemotherapy were combinations of etoposide and cisplatin (22 patients) or docetaxel and cisplatin (13 patients). Other chemotherapeutic agents included weekly taxol (4 patients) or cisplatin (1 patient). The patient characteristics are summarized in Table 1.

**Table 1:** Baseline clinical characteristics of the study group

**Characteristics**	**Number of patients**	**Percentage (%)**
Number of patients	80	100
Age (years)		
Mean	58.5	
Range	38-70	
Gender		
Male	48	60.0
Female	32	40.0
KPS		
90-100	63	78.7
80	17	21.3
AJCC clinical stage		
IIIA	35	43.8
IIIB	45	56.2
Histology		
Adenocarcinoma	31	38.8
Squamous cell	43	53.8
Large cell carcinoma	3	3.7
Other	3	3.7
Tumor site (lobe)		
Upper & middle	57	71.2
Lower	23	28.8
Comorbidity		
COPD	7	8.8
Cardiovascular disease	3	3.7
Diabetes	3	3.7
Smoking history		
Nonsmoker	34	42.5
Current or ex smoker	46	57.5
Chemotherapy		
Yes	56	70
No	24	30
V20		
Mean	32.11	
Range	8.6-63.3	
V30		
Mean	29.5	
Range	6.6-48.5	
Weight loss ≥ 5%		
Yes	29	36.2
No	51	63.8

Abbreviations: *COPD:* chronic obstructive pulmonary disease, *KPS:* Karnofsky performance status.

### The incidence of RP

RP at 6 to 8 weeks after the end of 3D-CRT are shown in Table 2. At 6 to 8 weeks, RP score 1 or more occurred in 38 (47.5%) of the 80 patients evaluated, including Grade 1 in 28 patients (35%), Grade 2 in 7 patients (8.75%), Grade 3 in 2 patients (2.5%). One patient (1.2%) suffered RP of Grade 4 and died of aggravation.

**Table 2:** Scoring of the 80 patients evaluated for RP after treatment

**Score**^**#**^	**No. of patients**	**%**
0	42	52.5
1	28	35.0
2	7	8.8
3	2	2.5
4	1	1.2

^#^Radiation pneumonitis was defined as the development of pulmonary toxicity of score ≥ 1.

### Changes of TGF-β1

**Figure 1:**
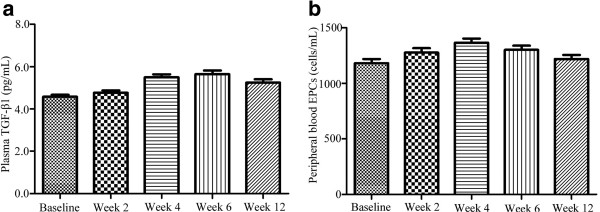
**Changes of TGF-β1 and EPCs during and after the treatment. a**. Change of TGF-β1 during and after the treatment. Variations of transforming growth factor-beta-1 (TGF-β1) at baseline, during, and after treatment in the whole group. Values are presented as mean ± S.E. **b**. Change of EPCs during and after the treatment. Variations of circulating endothelial progenitor cells (EPCs) at baseline, during, and after treatment in the whole group. Values are presented as mean ± S.E.

**Figure 2:**
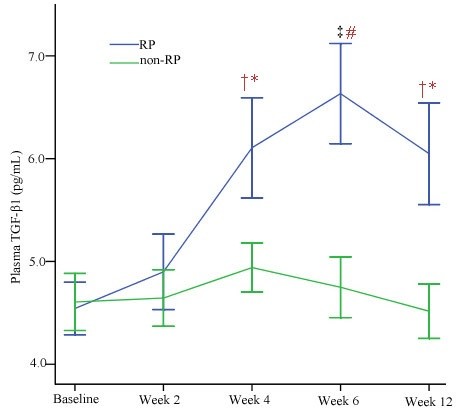
**Change of TGF-β1 during and after the treatment.** Variations of transforming growth factor-beta-1 (TGF-β1) at baseline, during, and after treatment in the RP group and the non-RP group. Values are presented as mean ± S.E. ^‡^*P* < 0.01 versus the baseline; ^†^*P* < 0.05 versus the baseline; ^#^*P* < 0.01 versus the control group; *P < 0.05 versus the control group.

The TGF-β1 levels at baseline, during, and after 3D-CRT were shown in Figure 1a. At baseline, the median TGF-β1 concentrations for RP patients and non-RP patients were 4.38 ng/ml (range, 3.24-6.23 ng/ml) and 4.30 ng/ml (range, 3.11-6.35 ng/ml), respectively. There was no significant difference between patients with and without RP (Table 3). During the period of 3D-CRT, the TGF-β1 levels in the RP group tended to increase significantly and reached a peak at 6 weeks. While in the non-RP group, the levels of TGF-β1 tended to increase relative to their pretreatment level, but no significant difference was found (Figure 2). We also performed an ANOVA model for repeated-measures for analysis of chronological changes in TGF-β1 levels and found that there were significant correlations between the TGF-β1 levels during the time course of 3D-CRT and the risk of developing RP.

**Table 3:** Comparison of TGF-β1 and EPCs at baseline in patients with and without RP

**Levels at baseline**	**RP**	**No RP**	P^*^
	**Mean SD**	**Mean SD**	
TGF-β1, ng/ml	4.54 ± 0.78	4.60 ± 0.89	0.73
EPCs, cells/ml	1201.4 ± 311.70	1164.6 ± 349.90	0.62

Abbreviations: *RP:* radiation pneumonitis, *SD:* standard deviation, *TGF-*β*1:* transforming growth factor-beta-1, *EPCs:* endothelial progenitor cells.

^*^ Independent-samples T test.

### Changes of EPCs

**Figure 3:**
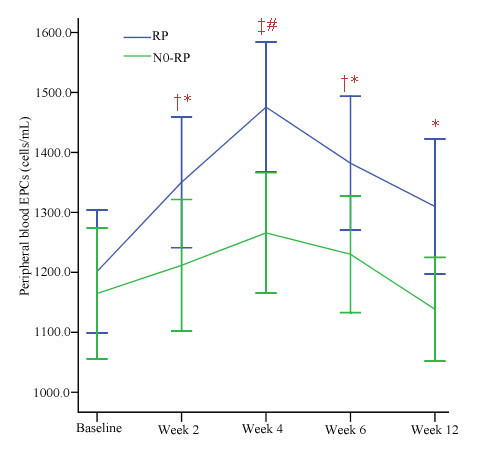
**Change of EPCs during and after the treatment.** Variations of endothelial progenitor cells (EPCs) at baseline, during, and after treatment in the RP group and the non-RP group. Values are presented as mean ± S.E. ^‡^*P* < 0.01 versus the baseline; ^†^*P* < 0.05 versus the baseline; ^#^*P* < 0.01 versus the control group; *P < 0.05 versus the control group.

We determined the number of EPCs (CD34+ cells) in the peripheral blood with flow cytometry. At baseline, the number of EPCs in RP patients was similar to that in the non-RP patients (1201.4 ± 311.7 cells/ml vs 1164.6 ± 349.9 cells/ml) (Table 3). The number of circulating EPCs at baseline, during, and after 3D-CRT did not vary significantly (Figure 1b). The analysis of variance showed that the occurrence of RP was significantly correlated with the variation of EPCs levels during 3D-CRT. As shown in Figure 3, the EPCs numbers in both RP patients and non-RP patients reached a peak at 4 weeks, and the difference of EPCs levels between patients who developed RP and those who did not was statistically significant during and after 3D-CRT.

### Association between the biomarkers and the clinical diagnosis of RP

We made statistical calculations and found that there was positive correlation between EPCs and the diagnosis of RP (r = 0.25, *P* < 0.01) and TGF-β1 and the diagnosis of RP (r = 0.55, *P* < 0.01). We also analyzed the correlation between the changes of EPCs and TGF-β1 in the same patient and found that there was a positive correlation between them (r = 0.12, *P* < 0.05).

### Clinical, functional, and dosimetric factors are predictive for RP at 6 to 8 weeks

The results concerning the predictive impact of clinical, functional and dosimetric factors have been shown in Table 4. In the univariate analysis, except for weight loss ≥ 5% (P = 0.05), no other clinical or functional factors at the baseline were significantly associated with the occurrence of RP. However, many dosimetric factors showed an association with RP. For the lung as a paired organ, V20 and V30 at the time of inclusion were statistically significant factors for the occurrence of RP (*P* = 0.02, and *P* = 0.001, respectively). MLD was also significantly associated with RP (*P* = 0.008).

**Table 4:** Clinical, functional, and dosimetric factors on RP

**Characteristics**	**RP**	**No-RP**	***P***
Age Mean (range)	58.5 (38–70)	59 (40–70)	0.83
Gender (Male/Female)	25/13	23/19	0.36
KPS Mean (range)	90.5 (80–100)	91.9 (80–100)	0.40
AJCC clinical stage (IIIA/ IIIB)	18/20	17/25	0.53
Histology (AD/SQ/LA/OT)	17/20/1/0	14/23/2/3	0.30
Tumor site (UM/LO)	29/9	28/14	0.34
Comorbidity (COPD/CA/DI)	2/1/0	5/2/1	0.80
Smoking history (NO/CU)	20/18	14/28	0.81
Chemotherapy (Yes/ No)	30/8	26/16	0.10
V20 Mean (range)	38.2 (8.6-63.3)	29.6 (8.9-60.2)	0.02
V30 Mean (range)	33.9 (7.9-48.5)	25.6 (6.6-51.0)	0.001
DLCO (%) Mean (range)	87.4 (61.3-99.4)	85.7 (65.4-98.7)	0.53
FEV1 (%) Mean (range)	86.3 (63.6-99.0)	83.4 (66.6-99.4)	0.24
FVC (%) Mean (range)	90.9 (75.6-99.6)	89.5 (74.3-99.4)	0.38
MLD Mean (range)	16.7 (5.9-25.6)	14.0 (7.9-21.3)	0.008
Weight loss ≥ 5% (Yes/ No)	18/20	11/31	0.0

Abbreviations: *KPS:* Karnofsky performance status, *AD:* Adenocarcinoma, *SQ:* Squamous cell, *LA:* Large cell carcinoma, *OT:* Other, *UM:* Upper&middle, *LO:* Lower, *COPD:* chronic obstructive pulmonary disease, *CA:* Cardiovascular disease, *DI:* diabetes, *NO:* Nonsmoker, *CU:* Current or ex smoker, *V20:* Percentage of irradiated lung volume exceeding 20 Gy, *V30:* Percentage of irradiated lung volume exceeding 30 Gy, *DLCO:* Diffusion capacity for carbon monoxide, *FEV1:* Forced expiratory volume at 1 second, *FVC:* Forced vital capacity, *MLD:* mean lung dose.

## Discussion

RP is a serious and potentially lethal treatment-related complication for lung cancer. The first finding of this study is that there was a high incidence (47.5%) of RP in NSCLC treated with 3D-CRT, which is higher than that in previous report [25]. The lack of consensus on uniform criteria for defining RP makes it difficult to diagnose, explain and compare the incidence and severity of RP. In approximately 28% of RP patients, the diagnosis is uncertain due to confounding factors [26]. In this article, we used a classification derived from the Lent-Soma scale defined by the RTOG and the EORTC [23]. This classification is based not only on clinical evaluation but also on measurements of loss of pulmonary function and radiological changes. In our study, the majority (35.0%) of RP patients suffered Grade 1, whereas 8.75% suffered Grade 2, 2.5% suffered Grade 3, and 1.2% suffered Grade 4. The high rate of RP observed in our study is thus mainly due to score 1 toxicity, which is similar to the results reported by other prospective studies using various scoring scales [27-29].

As all of us have known, the occurrence of pneumonitis is unpredictable. Therefore, reliable biochemical or cellular markers in identifying individuals at a high risk of developing RP are most desirable for early treatment modifications in order to avoid serious complications. These biomarkers may also allow the selection of patients who may be able to tolerate higher doses of radiation. Microvascular injury is a prominent feature of normal tissue radiation injury and plays a critical role in both acute (inflammatory) and chronic (fibrotic) radiation responses. Injury of the vascular endothelium is presumed to play a central role in the response of most normal tissues to ionizing radiation and to the progressive nature of chronic radiation fibrosis. This is particularly true for chronic radiation toxicity, in which microvascular injury seems to be a key to the unique self-perpetuating nature of radiation injury [30]. EPCs represent a subset of bone marrow derived cells that undergo mobilization secondary to a stimulus, circulate in the peripheral bloodstream where they home to sites of neovascularization, and differentiate into endothelial cells on site. Circulating EPCs may provide an endogenous repair mechanism to counteract ongoing risk factor-induced endothelial injury and therefore protect against the development of RP [31].

In our study, we first reported the correlation between the variations of circulating EPCs levels before, during and after 3D-CRT and the risk of RP in patients with NSCLC. We found that there was a statistically significant correlation between the variation of EPCs during and after 3D-CRT and the risk of developing RP, but no statistically significant correlation was found between the baseline level of EPCs and the risk of developing RP. EPCs levels began to increase in the first 2 weeks and reached a peak at week 4. EPCs levels were all significantly higher at week 2, week 4 and week 6 in patients with RP than that in patients without RP, respectively.

In acute situation, radiation causes endothelial cell apoptosis, increases vascular permeability, expression of chemokines and adhesion molecule. Others have recently demonstrated that vascular repair and reendothelialization after injury is enhanced by circulating EPCs [32,33]. Given these data, we speculate that 3D-CRT increases the mobilization of circulating EPCs, which may contribute to the repair of vascular and the enhancement of neoangiogenesis. Therefore, circulating EPCs levels are quantified to assess endothelial repair capacity and may be related to the rate of RP. Previous study has proved that irradiation can cause severe endothelium injury, which can start the mobilization of EPCs. On the other hand, endothelium injury also can induce extrinsic EPCs homing to the tissues [34]. Therefore, we presumed that the elevation of the number of circulating EPCs might represent a mechanism to enhance vascular health and reduce the damage of RP.

In this article, we have evaluated the utility of TGF-β1 as a predictor of RP. We found that the baseline levels of TGF-β1 in RP patients (4.38 ng/ml, range 3.24-6.23 ng/ml) were similar to that in non-RP patients (4.30 ng/ml, range 3.11-6.35 ng/ml). This finding is contrary to the result reported by Rübe [35]. The TGF-β1 levels during the period of 3D-CRT were significantly higher in patients with RP than that in patients without RP. We also found that the changes of TGF-β1 level during the course of 3D-CRT appeared to be useful in identifying patients at risk for developing RP. This finding is similar to the results by other researchers [36,37]. TGF-β1 plays a key role in tissue response to radiotherapy as a master switch for development and persistence of fibrosis [35]. In the lung parenchyma, TGF-β1 is synthesized by a large variety of cells, including platelets, leukocytes, and erythrocytes and its release into the plasma might be caused not only by the method of blood collection but also by pathological conditions of these cells. Therefore, there is conflicting data regarding the predictive ability of TGF-β1. Some clinical trials demonstrated that the incidence of RP was significantly correlated with plasma TGF-β1 level. The incidence of RP was significantly higher when TGF-β1 levels increased during radiotherapy or it failed to normalize after radiotherapy. Others also reported that there was a positive correlation between the levels of TGF-β1 before and during RT and the risk for developing RP [38].

Although TGF-β1 levels remained stable in the whole population during 3D-CRT, its circulating levels showed a statistically significant opposite evolution between patients with RP and patients without RP. TGF-β1 levels remained significantly elevated throughout the treatment in RP patients in comparison with that in non-RP patients, with a significant peak elevation at 6 weeks of treatment. TGF-β1 is a multifunctional cytokine involved in the regulation of immunologic and inflammatory response. The mechanisms through which TGF-β1 performs are complex, which involve both the inhibition of epithelial cell proliferation and the development of tissue fibrosis in response to irradiation. Radiation induces the release of TGF-β1 from a latent complex that can occur at radiation doses as low as 0.5 Gy [39]. TGF-β1 has multiple effects on vascular endothelial cells. It can induce angiogenesis [40]. Because TGF-β1 and EPCs mutually regulate vascular endothelial cells and can be considered in the complex interactive network, their levels during 3D-CRT may reflect the damage of vascular by radiation. In our study, the evolution of circulating TGF-β1 and EPCs levels during 3D-CRT was significantly associated with the risk of RP and seemed to be independent predictive factors for RP. This means that such variations could be an additional predictive tool to be used in association with dosimetric parameters and clinical character such as MLD, V20, V30 or weight loss ≥ 5% for a more precise evaluation of the risk of RP.

## Conclusion

In this study, we demonstrated that the variations of TGF-β1 and EPCs levels during 3D-CRT are significantly associated with the risk of RP. In the patients who developed RP, both TGF-β1 and EPCs level increased and reached a peak at 6 weeks and 4 weeks during 3D-CRT, respectively. On the contrary, the levels of TGF-β1 and EPCs in the patients who didn’t develop RP remain relative stable. Further research should be done to further identify biomarkers that might one day allow us to give rise to novel and specific prevention strategies for RP.

## Competing interests

The authors indicated no potential conflicts of interests.

## Authors' contributions

YL, TX and WZ were responsible for patient treatment and care. LZ collected the patients’ data, made figures and tables. XW performed all statistical analyses. YZ was responsible for the plasma TGF-β1 measurement and provided the information of measurement kit. YL and TX wrote the manuscript. WZ and YZ contributed to the analysis of data and revised the manuscript. HY conceived the study, helped to write and finalized the manuscript. All authors helped with the interpretation of the data, read and approved the final manuscript.

## References

[B1] Peulen H Karlsson K Lindberg K Tullgren O Baumann P Lax I Lewensohn R Wersäll P Toxicity after reirradiation of pulmonary tumours with stereotactic body radiotherapy Radiother Oncol 2011 101 2 260 266 10.1016/j.radonc.2011.09.012 22056534

[B2] Mazeron R Etienne-Mastroianni B Pérol D Arpin D Vincent M Falchero L Martel-Lafay I Carrie C Claude L Predictive factors of late radiation fibrosis: a prospective study in non-small cell lung cancer Int J Radiat Oncol Biol Phys 2010 77 1 38 43 10.1016/j.ijrobp.2009.04.019 20171801

[B3] Barthelemy-Brichant N Bosquée L Cataldo D Corhay JL Gustin M Seidel L Thiry A Ghaye B Nizet M Albert A Deneufbourg JM Bartsch P Nusgens B Increased IL-6 and TGF-beta1 concentrations in bronchoalveolar lavage fluid associated with thoracic radiotherapy Int J Radiat Oncol Biol Phys 2004 58 3 758 767 10.1016/S0360-3016(03)01614-6 14967431

[B4] Baker R Han G Sarangkasiri S DeMarco M Turke C Stevens CW Dilling TJ Clinical and dosimetric predictors of radiation pneumonitis in a large series of patients treated with stereotactic body radiation therapy to the lung Int J Radiat Oncol Biol Phys 2013 85 1 190 195 10.1016/j.ijrobp.2012.03.041 22929858

[B5] Saintigny P Burger JA Recent advances in non-small cell lung cancer biology and clinical management Discov Med 2012 13 71 287 297 22541616

[B6] Salama JK Vokes EE New radiotherapy and chemoradiotherapy approaches for non-small-cell lung cancer J Clin Oncol 2013 31 8 1029 1038 10.1200/JCO.2012.44.5064 23401449

[B7] Zhang HP Takayama K Su B Jiao XD Li R Wang JJ Effect of sunitinib combined with ionizing radiation on endothelial cells J Radiat Res 2011 52 1 1 8 10.1269/jrr.10013 21187670

[B8] Van der Meeren A Vandamme M Squiban C Gaugler MH Mouthon MA Inflammatory reaction and changes in expression of coagulation proteins on lung endothelial cells after total-body irradiation in mice Radiat Res 2003 160 6 637 646 10.1667/RR3087 14640783

[B9] Sabatier F Lacroix R Camoin-Jau L Anfosso F Sampol J Dignat-George F Circulating endothelial cells, microparticles and progenitors: towards the definition of vascular competence Rev Med Interne 2011 32 1 54 63 10.1016/j.revmed.2010.03.341 20541851

[B10] Ribatti D The involvement of endothelial progenitor cells in tumor angiogenesis J Cell Mol Med 2004 8 3 294 300 10.1111/j.1582-4934.2004.tb00319.x 15491505 PMC6740146

[B11] Thal MA Krishnamurthy P Mackie AR Hoxha E Lambers E Verma S Ramirez V Qin G Losordo DW Kishore R Enhanced angiogenic and cardiomyocyte differentiation capacity of epigenetically reprogrammed mouse and human endothelial progenitor cells augments their efficacy for ischemic myocardial repair Circ Res 2012 111 2 180 190 10.1161/CIRCRESAHA.112.270462 22589372 PMC3406600

[B12] Giannoni E Taddei ML Parri M Bianchini F Santosuosso M Grifantini R Fibbi G Mazzanti B Calorini L Chiarugi P EphA2-mediated mesenchymal-amoeboid transition induced by endothelial progenitor cells enhances metastatic spread due to cancer-associated fibroblasts J Mol Med (Berl) 2013 91 1 103 115 10.1007/s00109-012-0941-9 22903544

[B13] Yin M Liao Z Yuan X Guan X O'Reilly MS Welsh J Wang LE Wei Q Polymorphisms of the vascular endothelial growth factor gene and severe radiation pneumonitis in non-small cell lung cancer patients treated with definitive radiotherapy Cancer Sci 2012 103 5 945 950 10.1111/j.1349-7006.2012.02229.x 22320189 PMC3337362

[B14] Fosslien E Cancer morphogenesis: role of mitochondrial failure Ann Clin Lab Sci 2008 38 4 307 329 18988924

[B15] Zhang XJ Sun JG Sun J Ming H Wang XX Wu L Chen ZT Prediction of radiation pneumonitis in lung cancer patients: a systematic review J Cancer Res Clin Oncol 2012 138 12 2103 2116 10.1007/s00432-012-1284-1 22842662 PMC11824206

[B16] Rödel F Hantschel M Hildebrandt G Schultze-Mosgau S Rödel C Herrmann M Sauer R Voll RE Dose-dependent biphasic induction and transcriptional activity of nuclear factor kappa B (NF-kappaB) in EA.hy.926 endothelial cells after low-dose X-irradiation Int J Radiat Biol 2004 80 2 115 123 10.1080/09553000310001654701 15164793

[B17] Balzarini P Benetti A Invernici G Cristini S Zicari S Caruso A Gatta LB Berenzi A Imberti L Zanotti C Portolani N Giulini SM Ferrari M Ciusani E Navone SE Canazza A Parati EA Alessandri G Transforming growth factor-beta1 induces microvascular abnormalities through a down-modulation of neural cell adhesion molecule in human hepatocellular carcinoma Lab Invest 2012 92 9 1297 1309 10.1038/labinvest.2012.94 22732936

[B18] Luo H Zhang Y Zhang Z Jin Y The protection of MSCs from apoptosis in nerve regeneration by TGFβ1 through reducing inflammation and promoting VEGF-dependent angiogenesis Biomaterials 2012 33 17 4277 4287 10.1016/j.biomaterials.2012.02.042 22425554

[B19] Anitua E Troya M Orive G Plasma rich in growth factors promote gingival tissue regeneration by stimulating fibroblast proliferation and migration and by blocking transforming growth factor-β1-induced myodifferentiation J Periodontol 2012 83 8 1028 1037 10.1902/jop.2011.110505 22145805

[B20] Evrard SM d'Audigier C Mauge L Israël-Biet D Guerin CL Bieche I Kovacic JC Fischer AM Gaussem P Smadja DM The profibrotic cytokine transforming growth factor-β1 increases endothelial progenitor cell angiogenic properties J Thromb Haemost 2012 10 4 670 679 10.1111/j.1538-7836.2012.04644.x 22284809

[B21] Yu HM Liu YF Yu JM Liu J Zhao Y Hou M Involved-field radiotherapy is effective for patients 70 years old or more with early stage non-small cell lung cancer Radiother Oncol 2008 87 1 29 34 10.1016/j.radonc.2008.01.008 18237795

[B22] ICRU-50 Prescribing, Recording, Reporting, Photon Beam Therapy 1994 Washington, DC: International Commission on Radiation Units and Measurements

[B23] LENT SOMA tables Radiother Oncol 1995 35 17 60 7569012

[B24] Yu H Liu Y Han J Yang Z Sheng W Dai H Wang Y Xia T Hou M TLR7 regulates dendritic cell-dependent B-cell responses through BlyS in immune thrombocytopenic purpura Eur J Haematol 2011 86 1 67 74 10.1111/j.1600-0609.2010.01534.x 21039888

[B25] Ebara T Kawamura H Kaminuma T Okamoto M Yoshida D Okubo Y Takahashi T Kobayashi K Sakaguchi H Ando Y Nakano T Hemithoracic intensity-modulated radiotherapy using helical tomotherapy for patients after extrapleural pneumonectomy for malignant pleural mesothelioma J Radiat Res 2012 53 2 288 294 10.1269/jrr.11130 22374401

[B26] Yirmibesoglu E Higginson DS Fayda M Rivera MP Halle J Rosenman J Xie L Marks LB Challenges scoring radiation pneumonitis in patients irradiated for lung cancer Lung Cancer 2012 76 3 350 353 10.1016/j.lungcan.2011.11.025 22230037 PMC4287383

[B27] Kwa SL Lebesque JV Theuws JC Radiation pneumonitis as a function of mean lung dose: An analysis of pooled data of 540 patients Int J Radiat Oncol Biol Phys 1998 42 1 1 9 9747813 10.1016/s0360-3016(98)00196-5

[B28] Hernando ML Marks LB Bentel GC Radiation-induced pulmonary toxicity: A dose volume histogram analysis in 201 patients with lung cancer Int J Radiat Oncol Biol Phys 2001 51 3 650 659 10.1016/S0360-3016(01)01685-6 11597805

[B29] Martel MK Ten Haken RK Hazuka MB Dose-volume histogram and 3-D treatment planning evaluation of patients with pneumonitis Int J Radiat Oncol Biol Phys 1994 28 3 575 581 10.1016/0360-3016(94)90181-3 8113100

[B30] Cappuccini F Eldh T Bruder D Gereke M Jastrow H Schulze-Osthoff K Fischer U Köhler D Stuschke M Jendrossek V New insights into the molecular pathology of radiation-induced pneumopathy Radiother Oncol 2011 101 1 86 92 10.1016/j.radonc.2011.05.064 21722981

[B31] Cho HJ Kim HS Lee MM Kim DH Yang HJ Hur J Hwang KK Oh S Choi YJ Chae IH Oh BH Choi YS Walsh K Park YB Mobilized endothelial progenitor cells by granulocyte-macrophage colony-stimulating factor accelerate reendothelialization and reduce vascular inflammation after intravascular radiation Circulation 2003 108 23 2918 2925 10.1161/01.CIR.0000097001.79750.78 14568896

[B32] Kong Z Li J Zhao Q Zhou Z Yuan X Yang D Chen X Dynamic compression promotes proliferation and neovascular networks of endothelial progenitor cells in demineralized bone matrix scaffold seed Appl Physiol 2012 113 4 619 626 10.1152/japplphysiol.00378.2011 22723630

[B33] Xia WH Yang Z Xu SY Chen L Zhang XY Li J Liu X Qiu YX Shuai XT Tao J Age-related decline in reendothelialization capacity of human endothelial progenitor cells is restored by shear stress Hypertension 2012 59 6 1225 1231 10.1161/HYPERTENSIONAHA.111.179820 22547440

[B34] Zeng L Ding S Yan Z Chen C Sang W Cao J Cheng H Xu K Irradiation induces homing of donor endothelial progenitor cells in allogeneic hematopoietic stem cell transplantation Int J Hematol 2012 95 2 189 197 10.1007/s12185-011-1000-y 22258715

[B35] Rübe CE Palm J Erren M Fleckenstein J König J Remberger K Rübe C Cytokine plasma levels: reliable predictors for radiation pneumonitis? PLoS One 2008 3 8 e2898 10.1371/journal.pone.0002898 18682839 PMC2483418

[B36] Anscher MS Targeting the TGF-beta1 pathway to prevent normal tissue injury after cancer therapy Oncologist 2010 15 4 350 359 10.1634/theoncologist.2009-S101 20413640 PMC3227962

[B37] Anscher MS Thrasher B Zgonjanin L Rabbani ZN Corbley MJ Fu K Sun L Lee WC Ling LE Vujaskovic Z Small molecular inhibitor of transforming growth factor-beta protects against development of radiation-induced lung injury Int J Radiat Oncol Biol Phys 2008 71 3 829 837 10.1016/j.ijrobp.2008.02.046 18411002

[B38] Yu HM Liu YF Cheng YF Hu LK Hou M Effects of rhubarb extract on radiation induced lung toxicity via decreasing transforming growth factor-beta-1 and interleukin-6 in lung cancer patients treated with radiotherapy Lung Cancer 2008 59 2 219 226 10.1016/j.lungcan.2007.08.007 17870203

[B39] Choi CP Kim YI Lee JW Lee MH The effect of narrowband ultraviolet B on the expression of matrix metalloproteinase-1, transforming growth factor-beta1 and type I collagen in human skin fibroblasts Clin Exp Dermatol 2007 32 2 180 185 10.1111/j.1365-2230.2006.02309.x 17137474

[B40] Wang J Wang Y Wang Y Ma Y Lan Y Yang X Transforming Growth Factor β-regulated MicroRNA-29a Promotes Angiogenesis through Targeting the Phosphatase and Tensin Homolog in Endothelium J Biol Chem 2013 288 15 10418 10426 10.1074/jbc.M112.444463 23426367 PMC3624424

